# Serum human chorionic gonadotropin is associated with angiogenesis in germ cell testicular tumors

**DOI:** 10.1186/1756-9966-28-120

**Published:** 2009-08-27

**Authors:** Oscar Arrieta, Rosa Mayela Michel Ortega, Julián Ángeles-Sánchez, Cynthia Villarreal-Garza, Alejandro Avilés-Salas, José G Chanona-Vilchis, Elena Aréchaga-Ocampo, Arturo Luévano-González, Miguel Ángel Jiménez, José Luis Aguilar

**Affiliations:** 1 Department of Medical Oncology, Instituto Nacional de Cancerología, Mexico City, Mexico; 2 Experimental Oncology Laboratory, Instituto Nacional de Cancerología, Mexico City, Mexico; 3 Universidad Nacional Autónoma de México, Mexico City, Mexico; 4 Division of Surgical Oncology, Instituto Nacional de Cancerología, Mexico City, Mexico; 5 Department of Medical Oncology, Instituto Nacional de Ciencias Medicas y Nutrición, Mexico City, Mexico; 6 Department of Pathology, Instituto Nacional de Cancerología, Mexico City, Mexico; 7 Division of Urology, Instituto Nacional de Cancerología, Mexico City, Mexico

## Abstract

**Background:**

Germ cell testicular tumors have survival rate that diminishes with high tumor marker levels, such as human chorionic gonadotropin (hCG). hCG may regulate vascular neoformation through vascular endothelial growth factor (VEGF). Our purpose was to determine the relationship between hCG serum levels, angiogenesis, and VEGF expression in germ cell testicular tumors.

**Methods:**

We conducted a retrospective study of 101 patients. Serum levels of hCG, alpha-fetoprotein (AFP), and lactate dehydrogenase were measured prior to surgery. Vascular density (VD) and VEGF tissue expression were determined by immunohistochemistry and underwent double-blind analysis.

**Results:**

Histologically, 46% were seminomas and 54%, non-seminomas. Median follow-up was 43 ± 27 months. Relapse was present in 7.5% and mortality in 11.5%. Factors associated with high VD included non-seminoma type (*p *= 0.016), AFP ≥ 14.7 ng/mL (*p *= 0.0001), and hCG ≥ 25 mIU/mL (*p *= 0.0001). In multivariate analysis, the only significant VD-associated factor was hCG level (*p *= 0.04). When hCG levels were stratified, concentrations ≥ 25 mIU/mL were related with increased neovascularization (*p *< 0.0001). VEGF expression was not associated with VD or hCG serum levels.

**Conclusion:**

This is the first study that relates increased serum hCG levels with vascularization in testicular germ cell tumors. Hence, its expression might play a role in tumor angiogenesis, independent of VEGF expression, and may explain its association with poor prognosis. hCG might represent a molecular target for therapy.

## Background

Testicular cancer is a clinically, epidemiologically, and histologically heterogeneous group of neoplasms that represents 1% of malignant tumors in males. Germ cell testicular cancer is the most common type of tumor in males between 15 and 40 years of age, comprising approximately 98% of all testicular cancers, with an annual incidence of 7.5 per 100,000 inhabitants [1-3]. Germ cell testicular tumors are classified into two major sub-groups based on histological findings: seminomas and non-seminomas, each comprising approximately 50% of cases.

This malignancy possesses a high cure rate in its early and even in its metastatic stages, reaching 10-year survival rates between 90 and 100% [4,5]. However, there remains a sub-group of patients with poor prognosis with approximately 40% of 10-year mortality, regardless of treatment. In addition, 20–30% of germ cell tumors show recurrence that frequently exhibits refractoriness to multi-agent chemotherapy.

Human chorionic gonadotropin (hCG), alpha-fetoprotein (AFP), and lactate dehydrogenase (LDH) are serum tumor markers (STMs) that play a clear role in diagnosis, staging, risk classification, and clinical management of testicular germ cell tumors. Elevation of one or more markers is associated with disease progression and adverse prognosis [6,7]. Seminoma tumors do not increase AFP levels, and occasionally increase hCG [8].

One main feature of cancer is marked angiogenesis, which is essential for tumor growth and metastasis, exerting an impact on outcome and survival rates, including those of germ cell testicular tumors. The most important angiogenic stimulatory factor is vascular endothelial growth factor (VEGF), a mitogen specific for vascular endothelial cells [9]. VEGF is known for its ability to induce vascular permeability, to promote endothelial proliferation as well as migration, and to act as a critical survival factor for endothelial cells [10]. VEGF mRNA and protein expression is significantly higher in germ cell testicular tumors than in normal testis, and this expression correlates with microvascular density within the tumor [11]. Moreover, it has been shown that VEGF expression is correlated with metastases in these tumors [12].

hCG is a well-characterized hormone primarily produced by placenta and by other normal and tumor tissues in small amounts [13]. It has been described not only as an important peptide hormone during implantation [14], but also as an angiogenic factor for uterine endothelial cells [15]. It has been found that hCG possesses a role in the angiogenic process *in vivo *and *in vitro *by increasing capillary formation and endothelial cell migration in a direct association with the quantity of hCG administered; also, hCG-induced neovascularization was similar to that produced by VEGF and basic fibroblastic growth factor (bFGF) [16]. In addition, it has been proposed that hCG could induce VEGF production in tissues such as placenta [17] and granulosa cells [18,19].

Elevated hCG expression in serum, urine, or tumor tissue is usually a sign of aggressive disease and poor prognosis in germ cell tumors [8]. It is found in 40–60% of non-seminomatous germ cell tumors and in 30% of seminoma germ cell tumors [20]. However, no direct association has been reported between hCG and angiogenesis in cancer. The objective of this study was to determine the relationship between hCG serum levels, angiogenesis, and VEGF expression in germ cell testicular tumors.

## Methods

### Experimental design and patients

With previous Institutional Research and Ethics Board approval, we conducted a retrospective analytical study at the Instituto Nacional de Cancerología in Mexico City. We studied the tumor tissue of 101 patients with a diagnosis of germ cell testicular cancer that underwent surgery between 1992 and 2002.

AFP (normal range: 0–8.5 ng/mL), hCG (normal range: 0–4 mIU/mL), and LDH (normal range: 119–213 UI/L) serum levels were performed in all patients prior to surgery and before receiving chemotherapy, for risk stratification and follow-up. These markers were determined by using routine automated analyzers in the Department of Clinical Chemistry and Serum Markers, Instituto Nacional de Cancerología. The hCG was measured using the SIEMENS IMMULITE 2000 which is a highly specific, solid-phase, two-site chemiluminiscent immunometric assay that measures intact hCG without nicked forms and free subunits (Siemens; Los Angeles, CA, USA). AFP was measured with SIEMENS IMMULITE 2000 (Siemens; Los Angeles, CA, USA) and LDH with SYNCHRON LX20 (Beckman Coulter; Fullerton, CA, USA). Abdominal computed tomography scan and conventional chest x-ray were performed for disease staging according to the AJCC system. A database was made containing the clinical variables of all patients including IGCCCG risk status classification. Patients who received chemotherapy, radiotherapy, or both previous to surgery were excluded.

### Tissue retrieval and immunohistochemistry assays

Initial diagnostic biopsies were fixed in 10% neutral buffered formalin and embedded in paraffin. Morphologic evaluation was made in 3-μm tissue sections stained by the standard hematoxylin-eosin method. Sections 3-μm in thickness were mounted on slides and subsequently deparaffinized and rehydrated. Antigen was retrieved with 10 mM sodium citrate solution (pH 6.0) preheated to 80°C, maintaining this temperature and keeping sections in this solution for 10 min in a microwave pressure cooker. After allowing the sections to cool to room temperature, the slides were rinsed in PBS (pH 7.4). Endogenous peroxidase activity was blocked by incubation of the tissue samples for 10 min in 3% hydrogen peroxide. Samples were incubated for 45 min with the primary antibodies at room temperature in a moisture chamber.

### VEGF determination and analysis

Samples were incubated with mouse anti-VEGF monoclonal antibody (1:100) (Abcam, Cambridge MA, USA) in BSA 1% in PBS for 45 min. After washing with PBS, binding of the primary antibodies was revealed by incubation for 20 min with LSAB+ System Link (DAKO, Carpinteria, CA, USA) and LSAB+ HRP, (Streptavidin HRP kit, DAKO). The slides were rinsed with PBS and exposed to diaminobenzidine for 5 min. After washing with PBS and counter-staining with hematoxylin, the slides were dehydrated by graduated alcohols and xylol, and mounted with Poly-mount. Numerical proportions of stained cells were established by analyzing 10 high-power fields (400×) in each section. Only cytoplasmic staining was considered positive. Intensity was graded on a semi-quantitative scale from 0–3. Graduation of expression was considered negative if fewer than 5% of cells were stained.

### Determination of vascular density

The samples were incubated for 45 min with mouse anti-CD34 monoclonal antibody (1:200) (Biocare Medical, Concord, CA, USA) as a marker for vascular endothelial cells. Three separated, highly vascularized areas ("hot spots"), previously identified in high-power fields (100×, then 400×), were analyzed by two pathologists by means of optic microscopy without previous knowledge of hCG determinations. Any immunostained vessel clearly separated from adjacent vessels with no muscular wall and within the optic field was considered a neovascularization vessel. Vascular density (VD) was considered as the average of the three evaluated zones.

### Statistical analysis

For descriptive purposes, continuous variables were summarized as arithmetic means, medians, and standard deviations (SDs), while categorical variables were expressed as proportions and confidence intervals (CIs). Inferential comparisons were carried out using the Student *t *or the Mann-Whitney *U *test, according to data distribution determined by the Kolmogorov-Smirnov test. Chi square or Fisher exact test was used to assess significance between categorical variables. Statistically significant and borderline-significant variables (*p *< 0.1) were included in the multivariate logistic regression analysis. Overall survival time was measured from day of surgery to date of death or last follow-up visit and analyzed with the Kaplan-Meier method, and comparisons among sub-groups were performed with the log-rank test. For survival curve analysis, all variables were dichotomized. Adjustment for potential confounders was performed by multivariate regression analysis. Statistical significance was determined as *p *< 0.05 with a two-sided test. SPSS software package version 16 (SPSS Inc., Chicago, IL, USA) was employed to analyze the data.

## Results

A total of 109 patients were included. Two patients were excluded due to insufficient biopsy material and six because a different method to measure hCG was used. General patient characteristics are shown in Table 1. From a total of 101 tumors, non-seminomas corresponded to 54%, and seminomas to 46%. Diagnosis was confirmed by the pathologists, independent of the general characteristics of the patients. The most frequent histological sub-types were endodermal sinus tumors and mature teratoma in 21.8 and 14.9% of cases, respectively. Median age was 26 ± 7.7 years. The majority of patients (70.7%) had good risk according to the international risk (IGCCCG). hCG median and mean serum levels were 25.0 (range, 0–479000) and 14772 ± 71503, respectively. Only 10% of patients had hCG levels >5,000 mIU/mL, as shown in Table 2, percentiles for hCG, AFP and DHL values are also stated in this table.

**Table 1:** Patient characteristics (101 patients)

Characteristic	%	Median ± SD
Age (years)		26 ± 7.7

Histology		
Seminoma	46	
Non-seminoma	54	
Endodermal sinus	21.8	
Choriocarcinoma	5.0	
Embryonal cell carcinoma	8.9	
Mature teratoma	14.9	
Immature teratoma	2.0	
Teratocarcinoma	1.0	

TNM stage		
I	46.5	
II	27.3	
III	26.3	

Metastasis (N or M)		
Absent	48.5	
Present	51.5	

International consensus risk		
Good	70.7	
Intermediate	16.2	
Poor	13.1	

SD = standard deviation; TNM = Tumor, Node, Metastasis

**Table 2:** Serum tumor markers prior to surgery (101 patients)

Serum tumor markers	%	Mean ± SD	25%	50% (min-max)	75%	90%	95%	97.5%
AFP (ng/mL)		1214.3 ± 5892.2	1.85	14.7 (0–53800)	307.5	1748.6	5924.9	14182.0
≤1,000	89.1							
1,000–10,000	8.9							
≥10,000	2.0							

hCG (mIU/mL)		14772 ± 71503	0.0	25.0 (0–479000)	271.0	5000.0	66446.0	352040.0
≤5,000	90.1							
5,000–50,000	5.0							
≥50,000	5.0							

LDH (IU/L)		834 ± 929.1	253.5	475.0 (37–4568)	1070.0	1975.3	3247.2	4156.7
<1.5 × N	31.5							
1.5–10 × N	59.8							
>10 × N	8.7							

SD = standard deviation; AFP = alpha-fetoprotein; hCG = human chorionic gonadotropin; LDH = lactate dehydrogenase

**Figure 1:**
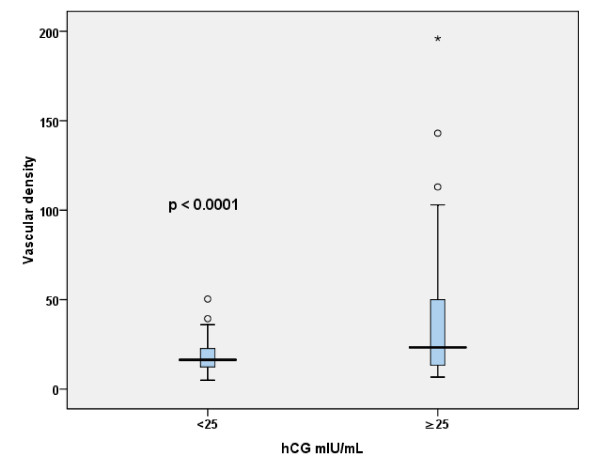
**Relationship between tissue vascular density and human chorionic gonadotropin (hCG) serum levels**.

Vascular density (VD) was determined in all samples. Median VD was 19.0 ± 28.9 (95% Confidence interval [95% CI], 5–75). Factors associated with higher VD were the following: AFP serum levels >14.7 ng/mL (*p *= 0.0001); serum hCG levels ≥ 25 mIU/mL (*p *= 0.0001), and non-seminomatous histologic type (*p *= 0.016) (Table 3 and 4). However, the sole factor independently related with VD was hCG elevation above the median (*p *= 0.04) (Table 5). When hCG levels were divided as <25 and ≥ 25 mIU/mL, we found that the latter were related with an increase in vascular neoformation (*p *= 0.0001) (Figure 1).

**Table 3:** Factors associated with vascular density

Variable	VD Mean ± SD	*p*
Age (years)		0.434
<26	30.58 ± 25.57	
>26	26.02 ± 31.29	

AFP (ng/mL)		0.0001
<14.7	17.23 ± 10.39	
≥14.7	38.57 ± 36.52	

LDH (IU/L)		0.092
<475	23.43 ± 24.61	
>475	34.01 ± 34.09	

hCG (mIU/mL)		0.0001
<25	18.27 ± 9.04	
>25	37.93 ± 37.7	

TNM		
I	23.84 ± 24.49	0.876 I vs. II
II	22.99 ± 18.49	0.024 I vs. III
III	41.49 ± 40.55	0.036 II vs. III

Metastases (N or M)		0.103
Absent	23.31 ± 24.10	
Present	32.88 ± 32.75	

SD = standard deviation; AFP = alphafetoprotein; hCG = human chorionic gonadotropin; LDH = lactate dehydrogenase; TNM = tumor, nodes, metastasis.

**Table 4:** Association of type of germ cell tumor with hCG levels and vascular density

Variable	hCG median (mIU/mL) ± SD	*p*	Vascular density ± SD	*p*
Seminoma	792.73 ± 2962.1	0.069	20.64 ± 20.14	0.016

Non-seminoma	26954 ± 96511.2		34.56 ± 33.70	

hCG = human chorionic gonadotropin; SD = standard deviation

**Table 5:** Multivariate analysis of factors associated with vascular density

Variable	Regression co-efficient	*p*
Histology (S vs. NS)	0.2	0.907

Metastatic disease	1.2	0.165

hCG	14	0.04

AFP	13.4	0.08

LDH	0.73	0.92

S = seminoma; NS = non-seminoma; hCG = human chorionic gonadotropin; AFP = alpha-fetoprotein; LDH = lactate dehydrogenase

VEGF expression was determined in 57 biopsies due to insufficient material. Its expression was present in 56% of the samples. Average percentage of expression was 19 ± 3% (minimum, 0%; maximum, 80%). Intensity was absent in 44%, mild in 48%, and moderate in 8%. Qualitative VEGF expression and expression intensity were not associated with either VD or hCG serum levels (Table 6).

**Table 6:** Association of VEGF expression with hCG levels and vascular density

Variable	hCG median (mIU/mL) ± SD	*p*	Vascular density median ± SD	*p*
VEGF		0.422		0.821
Absent	1840.7 ± 4444.0		25.44 ± 26.61	
Present	16581.0 ± 85185.0		27.06 ± 23.72	

VEGF intensity		NS		NS
Absent	1840.7 ± 4444.7		25.44 ± 26.61	
Low	19337 ± 91973.8		28.43 ± 25.18	
Moderate	47.35 ± 71.86		18.83 ± 9.85	

VEGF = Vascular endothelial growth factor; hCG = human chorionic gonadotropin; SD = standard deviation

Median follow-up time was 43 ± 27 months. Recurrence was observed in 7.5% and death in 11.5% of patients. Disease-free survival (DFS) at 2 and 5 years was 93.7% (95% CI, 88–98) and 83% (95% CI, 68–98), respectively. By analyzing DFS-related factors, only high international risk correlated with worse prognosis (*p *= 0.005). VD and VEGF expression were not associated with recurrence.

## Discussion

hCG is considered an extremely sensitive and specific marker of germ cell testicular tumors. Its increased serum levels usually correlate with the existence of viable cancer cells and it is often associated with disease progression, recurrence, and a worse prognosis [7,21,22]. Therefore, hCG levels are part of the international risk factor assessment [23].

In the present study, hCG is associated with elevated VD in testicular tumors. hCG has been associated with angiogenesis in normal tissues; this has been confirmed *in vivo *and *in vitro *by increasing capillary formation and endothelial cell migration [16,18], and in regulation of placental angiogenesis [24]. Elevated hCG serum levels are present in pregnancy; thus, similarities between tumor invasion and its vascularization and blastocyst implantation and placental development have been described [25,26]. In addition, it has been proposed that hCG could induce VEGF production in tissues such as placenta [17] and granulosa cells [18,19]. hCG administration to women undergoing *in vitro *fertilization increases urinary [27], serum, and follicular-fluid VEGF concentrations [28]. Furthermore, hCG exerts a direct angiogenic effect on hCG/LH receptor-expressing uterine endothelial cells, which respond with increased capillary formation *in vitro *[16,29]. hCG receptors have been detected in breast carcinoma tissue, which indicates a probable link to a worse breast-cancer prognosis during pregnancy, which we previously hypothesized [30].

We found that predominantly in patients with hCG serum levels ≥ 25 mIU/mL there was increased tumoral vascular neoformation, suggesting that hCG could be involved in angiogenic processes during tumor development. Intrinsic hCG activity is clinically relevant when serum concentrations are high, for instance, during pregnancy or under certain pathological conditions that might be associated to the carcinogenesis of testicular germ cells [6,7].

In this study, a prominent VD (median, 19.0 ± 28.9) was observed in all tumors, especially non-seminomas, which would be expected as hCG is elevated in this subtype of germ tumors. Angiogenesis is essential for malignant neoplasm progression and is correlated with poor prognosis in numerous solid tumors [31], including germ cell testicular cancer [32,33]. Particularly in normal testis, the endothelial cell proliferation rate is considerably higher than in other stationary organs. It has been shown that this rate can be increased via hCG stimulation of Leydig cells [34]. In addition, a correlation between hCG and VEGF has been confirmed in rat models and transformed mouse Leydig cell lines (MA-10 cells) [35,36].

In our results, VEGF expression was limited to 56% of the tumors studied, showing no clinical or histopathological association; nevertheless, tissue availability comprised a factor that could render the data less significant. VEGF expression in germ cell testicular tumors was previously found to be significantly higher than in normal testis and was correlated with microvessel density [11,37]; it was also described as an indicator of metastatic disease [12]. However, another study reported no prognostic significance in relation to metastatic potential, sustaining the possibility of the existence of additional factors affecting metastatic capacity. It has also been suggested that there might be other angiogenic factors, different from VEGF, which are important in testis tumor biology [37].

No significant association was found between VD and VEGF expression or prognosis according to disease-free survival. This could be a consequence of the low recurrence rate in our population (70% of our patients presented a good international risk), making it difficult to find a statistical association. With similar results, in a study of 51 patients with stage I disease, no association was found between VD and VEGF expression and DFS [37]. Concerning these results, there is a possibility that angiogenic factors other than VEGF are relevant in the development of this neoplasm's vascularization, taking into account the fact that modulation of the angiopoietin family has been previously described in non-tumor models [38,39], as well as fibroblast growth factor [40], metalloprotease induction, and cellular adhesion-molecule expression [41].

Unexpectedly, we found no correlation between hCG serum levels and VEGF tissue expression. Our results indicate that hCG and VEGF may operate through different signaling pathways for angiogenesis stimulation, and suggest that hCG is not only an independent prognostic factor, but that also it additionally plays a role in the pathophysiology of these neoplasms, representing a potential therapeutic target in patients showing significant elevations of this hormone and who display no response to treatment.

## Conclusion

Our study shows that hCG elevation is independently associated with high VD in testicular germ cell tumors, but not with VEGF expression. This suggests that hCG plays an important function in the angiogenesis and pathophysiology of germ cell neoplasms, being a likely target of treatment by receptor inhibition, activity blockage, or obstruction of intracellular pathways it triggers.

## Competing interests

The authors declare that they have no competing interests.

## Authors' contributions

OA design and conception of the study, analysis of data, revision of the manuscript, RMM acquisition and analysis of data, draft and revision of the manuscript, JAS acquisition of data, CVG critically revised the manuscript and also contributed to the analysis, AAS supervised the immunohistochemistry, revised the manuscript, JGCV checked the immunohistochemistry, revised the final version, EAO revised the data, ALG carried out the immunohistochemistry, MAJ critical revision of the manuscript and JLA conception of the study and revision of the manuscript. All authors have read and approved the final version of the manuscript.
